# Health care professionals at risk of infection with Borna disease virus – evidence from a large hospital in China (Chongqing)

**DOI:** 10.1186/s12985-015-0239-y

**Published:** 2015-03-12

**Authors:** Xia Liu, Liv Bode, Liang Zhang, Xiao Wang, Siwen Liu, Lujun Zhang, Rongzhong Huang, Mingju Wang, Liu Yang, Shigang Chen, Qi Li, Dan Zhu, Hanns Ludwig, Peng Xie

**Affiliations:** Department of Neurology, The First Affiliated Hospital of Chongqing Medical University, 1 Yixueyuan Road, Yuzhong District, Chongqing, 400016 China; Shanghai Key Laboratory of Forensic Medicine, Institute of Forensic Science, Ministry of Justice, P.R. China, Shanghai, 200063, China; Institute of Neuroscience, Chongqing Medical University, Chongqing, 400016 China; Chongqing Key Laboratory of Neurobiology, Chongqing, 400016 China; Department of Rehabilitation, The Second Affiliated Hospital of Chongqing Medical University, Chongqing, 400016 China

**Keywords:** Borna disease virus, Risk of infection at hospital, Health care professionals, Major depressive disorder, Circulating immune complexes, RT-qPCR, China

## Abstract

**Background:**

Human Borna disease virus (BDV) infections have recently been reported in China. BDV causes cognitive and behavioural disturbances in animals. The impact on human mental disorders is subject to debate, but previous studies worldwide have found neuropsychiatric patients more frequently infected than healthy controls. A few isolates were recovered from severely depressed patients, but contagiousness of BDV strain remains unknown.

**Method:**

We addressed the risk of infection in health care settings at the first affiliated hospital of Chongqing Medical University (CQMU), located in downtown Chongqing, a megacity in Southwest China. Between February 2012 and March 2013, we enrolled 1529 participants, of whom 534 were outpatients with major depressive disorder (MDD), 615 were hospital personnel, and 380 were healthy controls who underwent a health check. Infection was determined through BDV-specific circulating immune complexes (CIC), RNA, and selective antibodies (blood).

**Results:**

One-fifth of the hospital staff (21.8%) were found to be infected (CIC positive), with the highest prevalence among psychiatry and oncology personnel, which is twice as many as were detected in the healthy control group (11.1%), and exceeds the prevalence detected in MDD patients (18.2%).

**Conclusion:**

BDV circulates unnoticed in hospital settings in China, putting medical staff at risk and warranting clarification of infection modes and introduction of prevention measures.

## Background

Major depressive disorder (MDD) is a debilitating psychiatric illness with a wide variation in lifetime prevalence across countries. Mean estimates are 14.6% in high- and 11.1% in low-middle income countries according to the World Mental Health Survey, with China (Shenzen) ranging among countries with the lowest lifetime estimates (6.5%) [1]. On a global scale, among mental and substance use disorders which account for one-fifth (22.9%) of all non-fatal burden of disease in 2010, depressive disorders contributed the most, accounting for 42.2% of years lived with disability (YLDs) [1]. The hypothesis that an ancient neurotropic virus, Borna disease virus (BDV), may contribute to MDD’s multi-factorial etiology has been supported by numerous studies demonstrating serological and molecular epidemiological evidence not only for human infection but for differences between psychiatric patients and healthy controls [2].

BDV (*Bornaviridae; Mononegavirales*), a non-cytolytic RNA virus, preferentially infects limbic structures and causes cognitive deficiencies and behavioral abnormalities in rodents [3]. Remarkably, human BDV strain Hu-H1, isolated from a severely depressed bipolar patient’s blood cells in Germany [4], promotes apoptosis and inhibits cell proliferation in vitro, whereas laboratory strain V dating back to a horse with fatal Borna disease (Germany, 1927) and having a history of multiple passaging (rabbits, rats, cell culture), does the opposite [5]. MDD patients display higher detection rates of BDV-specific RNA transcripts in lymphocytes, increased serum antibody titers against BDV-specific nucleoprotein (p40) and phosphoprotein (p24), and higher prevalence of BDV-specific circulating immune complexes (CIC) than healthy individuals [6-10]. The predominance of CIC to indicate active infection has recently been confirmed in 2014 by Mazaheri-Tehrani et al. who investigated clinically accurately diagnosed psychiatric patients and did detect a high infection (CIC) prevalence of 40.4% which was significantly different from that of healthy controls (29.5%; p = 0.036) [11]. However, using methods determining BDV-specific antibodies or nucleic acids, a recent study failed in respect of either psychiatric patients or control subjects [12]. Differing sensitivities in detection methods currecnly impair comparability of worldwide prevalence data. Among all markers/methods currently in use, BDV-CICs, not only are the most frequent indicators of virus infection, but also reasonably explain infection profiles of dormant and activated states, the latter characterized by antigen production, release into the plasma, host antibody response, and formation of CIC [2,7].

Regardless of flaws due to methods-driven discrepancies, the BDV hypothesis stays intriguing because it is fitting as well in other models like the concept of stress-induced depression [13], as hypothalamic-pituitary-adrenal (HPA) system activity in depressed patients has been associated with BDV but not with other infections [14].

The transmission mode of BDV is still elusive, but nasal, conjunctiva secretions, saliva and blood are likely sources [15]. In family members and mental healthcare workers of psychiatric patients, higher BDV infection prevalence has been reported in comparison with unrelated persons [16]. Whether hospital personnel are at risk of acquiring BDV infection through patients’ care is public health issue of considerable relevance, particularly in densely populated countries such as China, where BDV research is still scarce [17].

Here, we report a cross-sectional epidemiological survey of BDV infection including MDD outpatients, hospital personnel and healthy individuals who underwent a health check-up at the 3000-beds First Affiliated Hospital of Chongqing Medical University (CQMU) in Chongqing, a megacity of about 30 million inhabitants in Southwest China.

## Methods

### Ethics statement

All subjects gave their written informed consent after a detailed introduction to the study. Either of the parents of the minors gave their written informed consent on behalf of their participating child. Among the MDD patients 17 adolescents aged 15–17 years were included. The study was approved by the Ethics Committee of Chongqing Medical University (#2011002) and was performed according to the Helsinki Declaration.

### Study population

A total of 1529 participants were recruited at the above-mentioned hospital between February 2012 and March 2013, and assigned to the three groups described (Table 1).

**Table 1:** **Demographic variables and BDV infection prevalence in study participants**

	**I, MDD outpatients**	**II, Hospital personnel**	**III, Healthy, controls**	***P*** **-value**			
				**Group I vs. II**	**Group I vs. III**	**Group II vs. III**	**All three groups**
**Sample size**	534	615	380				
**Age (year)** ^**a, b**^	35.99 ± 12.23	39.28 ± 11.61	35.58 ± 10.86	0.000	0.885	0.000	0.000^e^
<25	116	38	57				
25-39	217	284	193				
40-54	147	226	105				
55-70	54	56	25				
>70	0	11	0				
**Sex (M/F)** ^**c**^	187/347	182/433	160/220	0.049	0.030	0.000	
**HDRS score** ^**a,d**^	20.58 ± 7.00	2.2 ± 0.8	1.2 ± 1.0	0.000	0.000	0.143	
**CIC+ (%)** ^**c**^	97 (18.2%)	134 (21.8%)	42 (11.1%)	0.001	0.024	0.000	
**p24 RNA+ (%)** ^**c**^	27 (5.1%),	50 (8.1%),	8 (2.1%),	0.038	0.022	0.000	
**CIC+ and p24 RNA +** ^**c**^	21 (3.9%),	48 (7.8%)	5 (1.3%)	0.006	0.019	0.000	
**CIC+ and/or p24 RNA +** ^**c**^	103 (19.3%),	136 (22.1%),	45 (11.8%),	0.239	0.003	0.000	
**CIC- and p24 RNA+**	6	2	3				
**CIC level**							0.197^f^
Negative and borderline	437	454	332				
Weakly/1+ positive	82	134	42				
2+ positive	14	23	6				
3+ and 4+ positive	1	4	0				
**Antibody+/CIC+**	5/50	3/50	4/40				
**Antibody+/CIC+**	0/50	1/50	1/40				

*Abbreviations*: *MDD* major depressive disorder, *M* male, *F* female, *HDRS* Hamilton Depression Rating Scale, *CIC* circulating immune complexes, *p24* BDV phosphoprotein 24, *RNA* ribonucleic acid. ^a^Values expressed as means ± SDs.,^b^Mann-Whitney test,^c^Chi-square test,^d^One-way ANOVA,^e^Kruskal-Wallis test,^f^Spearman correlation.

MDD outpatients were enrolled at the Psychiatric Center and ranked for severity according to DSM-IV criteria and the 17-item version of the Hamilton Depression Rating Scale (HDRS). A total of 534 MDD outpatients were included (males = 187, females = 347) aged 15 to 66 years (mean = 35.99 ± 12.23 years, median = 36 years). Patients with confounding factors, such as pre-existing physical or mental disorders other than MDD, medication, and/or illicit drug use, were excluded. A total of 615 hospital personnel members (males = 182, females = 433) aged 20 to 81 years (mean = 39.28 ± 11.61 years, median = 39 years) were included after accepting HDRS assessment. At the same time, 380 healthy controls (males = 160, females = 220) aged 21 to 64 years (mean = 35.58 ± 10.86 years, median = 33.50 years) were recruited from the Medical Examination Center. Candidates with any lifetime history of neurological, DSM-IV Axis I or II, and/or systemic illness were excluded.

### Plasma and peripheral blood mononuclear cell (PBMC) preparations

EDTA-treated blood samples (10 ml) were separated into PBMCs and plasma by Ficoll-Conray centrifugation (density, 1.087 g/ml) and stored at −80°C.

### CIC assays

Plasma-based BDV CIC was assayed as previously described and standardized [7]. Test specificity is based on two monoclonal antibodies (mAbs), BDV p40 (W1) and p24 (Kfu2) [18], binding to conformational epitopes on either N or phosphorylated P-protein including N/P heterodimers according to epitope mapping [19]. Sensitivity has been determined for W1 mAb using purified recombinant N-protein (limit 0.15-0.3 ng/100 μl/well) [19]. In brief, polystyrene 96-well plates (MaxiSorp; Nunc) were coated with anti-mouse IgG Fc (1:1000 for 1 h at 37°C, Jackson ImmunoResearch). After washing, BDV mAbs (hybridoma supernatant, 1:500 each for 1 h at 37°C) were added. Estimated total mAb concentration per well (0.2 μg) was equivalent to that of anti-mouse IgG, but exceeding 1000 times the detection limit for N-protein. This step included block proteins (fetal calf serum) in 200-fold excess. After washing, plasma samples (1:20 and serially two-fold dilution in PBS-T) were incubated for 1 h at 37°C. Serum sampled from a German MDD patient (CIC, optical density (OD) = 0.486 at 415 nm) and a healthy German individual (OD = 0.09) were used throughout all experiments as positive and negative controls, respectively. After washing, alkaline phosphatase-conjugated anti-human IgG (1:3000 in TBS-T, Jackson ImmunoResearch) was applied for 1 h at 37°C. After washing, freshly prepared substrate *p*-nitrophenylphosphate (pNPP, 1 mg/ml) in 1 M diethanolamine buffer (AP Substrate Kit, Bio-Rad) was added (5 min at room temperature, visual control), followed by a stop using 50 μl of 3 M sodium hydroxide. A Bio-Rad imarkMicroplate Reader was used at 415 nm. The OD levels of BDV CIC were scored as previously described [7]:

- negative, cut-off value (OD ≤ 0.10); borderline (OD > 0.10 – 0.12);
- weakly positive (OD >0.12 – 0.15); >
- 1+ positive (OD >0.15 – 0.30);
- 2+ positive (OD >0.30 – 0.6);
- 3+ positive (OD >0.60 – 1.00); and
- 4+ positive (OD >1.00).

Plasma samples determined to be positive by the initial EIA were tested a second time. All positive samples from the three groups (306/1529) were evenly distributed to each plate in the second round. Those samples that tested positive in both rounds (273/1529) were deemed positive.

### Antibody assays

BDV antibodies in blood plasma were determined as previously described [7], with slight modifications as follows: the first two steps as CIC assay including coating with Goat Anti-Mouse IgG and then with BDV p40 and p24 mouse monoclonal antibodies. After washing, ultrasonicated BDV strain Hu-H1 infected oligodendroglia (OL) cells (titer 1.1 × 10^4^ focus forming units ml ^−1^), diluted 1:50 in PBS-T, were incubated 1 h at 37°C. After washing, plasma samples, diluted 1:100 and serially two-fold in PBS-T, were incubated for 2 h at 37°C. The next steps were the same as for the CIC assay.

Due to limited volumes, only 280 plasma samples were additionally examined by the BDV antibody assay. Samples were randomly selected as follows: 50 plasma samples each from MDD patients and hospital staff with CIC positive and CIC negative results, as well as 40 samples each from healthy controls with CIC positive and CIC negative results.

### Reverse transcription quantitative PCR (RT-qPCR)

Total RNA was extracted from PBMCs using Trizol (Life Technologies) according to the manufacturer’s protocols, dissolved in 25 μl of DNase/RNase-free H_2_O, and stored at −80°C. RNAs from uninfected oligodendrocytes (negative control) and RNAs from BDV-infected oligodendrocytes (positive control) were simultaneously processed. RNA was reverse-transcribed into cDNA (Reverse Transcriptase System, Promega), then incubated with random primer and 30 units of reverse transcriptase for 60 min at 42°C. After enzyme inactivation (5 min at 95°C), the cDNA was stored at −20°C.

RT-qPCR was performed as previously described [20]. In brief, PCR amplification was performed in 25 μl per well, each containing 12.5 μl of 2 × TaqMan Universal PCR Master Mix with UNG (Applied Biosystems), 600 nM p24 primer, 160 nM p24 probe, and 3.5 μl of cDNA. The PCR steps were as follows: 2 min at 50°C to activate the uracil-N-glycosylase (UNG) that detects and degrades the former PCR products; 10 min at 95°C to inactivate UNG and activate the polymerase; 40 cycles of denaturation at 95°C for 15 s; annealing and elongation at 60°C for 1 min. All reactions were run in a Corbett Research Rotor-Gene 6000 thermocycler including a no-template control (sterile water) and repeated at least three times. Signals were regarded as positive only if the fluorescence intensity exceeded 10 times the standard deviation of the baseline fluorescence (threshold). Cq values greater than 40 were regarded as negative. Eukaryotic 18S rRNA (Applied Biosystems) was amplified as an endogenous control.

### Cloning and sequencing of RT-qPCR products

RT-qPCR products were cloned into the pGEM-T Easy vector system (Promega). One to three clones of each product were sequenced by the Applied Biosystems Prism dye-terminator dideoxy system at a commercial facility (BGI Life Tech. Beijing, China). Sequences were analyzed by comparison with GenBank.

## Results

### BDV-specific CIC

Of the 1529 individual samples analyzed by BDV-specific CIC EIA, 273 were positive (17.9%; 18.6% females (186/1000) and 16.4% males (87/529)). While CIC prevalence among the three groups was significantly different, CIC levels were not (*p* = 0.197, Spearman correlation; Table 1). Gender differences were not significant either (*p* = 0.296, Chi-Square test), in contrast to age (*p* = 0.030, Chi-Square Test) (Table 2). The trend of CIC prevalence increasing with age remained after adjustment to ages present in all three groups (Table 1).

**Table 2:** **Gender or Age and CIC prevalence in all three groups**

	**CIC-**	**CIC+**	**Totals**	**CIC+ %**
**Gender**				
**Male**	442	87	529	16.4%
**Female**	814	186	1000	18.6%
**Totals**	1256	273	1529	17.9%
**Age group**				
**<25**	173	38	211	18.0%
**25-39**	566	128	694	18.4%
**40-54**	375	103	478	21.5%
**55-70**	102	33	135	24.4%
**>70**	7	4	11	36.4%
**Totals**	1223	306	1529	20.0%

Prevalence in females compared to males was higher but not significant (Chi-Square tests: χ2 = 1.094, *p* = 0.296) CIC prevalence increases with age (Chi-Square tests: χ_linear2_ = 4.682, p = 0.030).

BDV-specific CICs were differentially detected in 18.2% (97/534) of MDD outpatients vs. 11.1% (42/380) of healthy controls (*p* = 0.024, Chi-Square test). The severity of depression, however, was neither related to CIC prevalence (*p* = 0.194, Chi-Square test) nor CIC levels (*p* = 0.600, Spearman’s correlation; Table 3). MDD outpatients and healthy controls were matched to age but not gender (age, *p* = 0.885, Mann–Whitney test; gender, *p* = 0.030, Chi-Square test; Table 1).

**Table 3:** **CIC prevalence or CIC levels and HDRS in MDD outpatients**

	**HDRS score**				
	**0-7**	**8-16**	**17-23**	**≥24**	**Totals**
**CIC prevalence**					
**CIC-**	26	53	218	140	437
**CIC+**	10	11	39	37	97
**Totals**	36	64	257	177	534
**CIC+ %**	27.8%	17.2%	15.2%	20.9%	18.2%
**CIC level**					
**Negative and borderline**	26	53	218	140	437
**Weakly positive**	3	5	11	7	26
**1+ positive**	6	6	21	23	56
**2+ positive**	1	0	6	7	14
**3+ positive**	0	0	1	0	1
**Totals**	36	64	257	177	534

BDV prevalence was not significantly related to the severity of depression (Chi-Square tests: χ2 = 4.718, *p* = 0.194). CIC levels were not significantly related to the severity of depression (Spearman’s correlation coefficient =0.023, *p* =0.600).

BDV-specific CICs were significantly more prevalent in hospital personnel (134/615) than in MDD outpatients (21.8% vs. 18.2%; *p* = 0.001, Chi-Square test) and healthy controls (21.8% vs. 11.1%, *p* = 0.000, Chi-Square test). Overall, no significant association between CIC prevalence and type of hospital department was found (*p* = 0.941, Chi-Square test), but a trend to slightly higher CIC prevalence was obseved among oncology and psychiatry personnel (*p* = 0.540, Chi-Square test) (Table 4).

**Table 4:** **CIC prevalence by hospital department**

**Department**	**CIC-**	**CIC+**	**Totals**	**CIC+ %**
**Oncology and psychiatry**	15	7	22	31.8%
**General/Internal medicine**	253	87	340	25.6%
**Medical laboratory**	60	23	83	27.7%
**Administrative staff logistics**	94	36	130	27.7%
**Not recorded**	32	8	40	20.0%
**Totals**	454	161	615	26.2%

No correlation between BDV infection and department distribution was found (Chi-Square tests: χ2 = 0.395, *p* = 0.941). A trend to a somewhat higher CIC prevalence at oncology and psychiatry compared to other departments (Chi-Square test, *p* = 0.540).

### BDV-specific antibodies

Of randomly selected MDD patients who had tested CIC-positive, 10% had antibodies (5/50), as opposed to none of CIC-negative patients (0/50). Of the hospital personnel, 6% (3/50) of the CIC-positive and 2% (1/50) of the CIC-negative tested positive for antibodies. In the healthy control group, 10% (4/40) of the CIC-positive and 2.5% (1/40) of the CIC-negative subjects tested positive for antibodies,

### BDV p24 RNA in PBMCs

Negative and positive controls performed appropriately in each RT-qPCR assay, with Cq values of BDV+ control samples less than 25 cycles. RNA quality of all samples was appropriate due to Cq values of 18S RNA which were between 20 and 25 cycles as well. Specificity of amplification products was demonstrated by DNA sequencing revealing a greater than 96% identity with GenBank’s BDV p24 fragments.

BDV p24 RNA was present in PBMCs from 5.1% of MDD outpatients (27/534), 8.1% of hospital personnel (50/615), and 2.1% of healthy controls (8/380). RNA+ individuals were not always CIC+. The differences across all groups were significant (Table 1).

## Discussion

This is the largest survey ever performed on human BDV infection, representatively conducted in a hospital setting of a Chinese megacity with high urban density (Chongqing). The survey involved more than 1500 subjects including comparable numbers of MDD patients, hospital staff, and controls. The study used two serological methods and one molecular biological method, determining BDV- CIC and antibodies (selected panel) in plasma and BDV-p24-RNA in PBMCs.

We applied the specificity-proven BDV CIC EIA here [19] to screen for the most prevalent plasma-based indicator of BDV infection [7]. As explained, CIC monitoring allows comparative prevalence analysis of infected subjects who undergo periods of virus activity accompanied by over-expression and shedding of BDV proteins (N/P) into the plasma which, in turn, leads to antibody generation and subsequent CIC formation [2,19]. Compared to CIC, the mean antibody prevalence in the Chinese healthy controls was significantly lower (5/80; 6.25%), although a selection bias of 50% CIC-positive samples was used. Our study supported previous findings [2] that through CIC formation antibodies become less frequent in the plasma and are inferior to CIC for estimating the prevalence of BDV infection. Based on CIC, BDV infection in healthy individuals is considerably less prevalent in Southwest China (11.1%) in comparison to European countries such as Germany (32.3%) [2,7] and the Czech Republic (37.3%) [21], or to Middle East countries like Iran (29.5%) [11], suggesting large regional differences. We also confirm previously described significant differences in CIC prevalence between MDD outpatients and healthy controls [2], thereby providing further evidence that BDV infection may play a role in MDD pathogenesis. This implies at the same time that infected healthy people are at risk, and increased CIC prevalence would indicate frequent activation.

The novelty of our approach is to address the risk of infection for medical staff in professional health care settings, where MDD patients, hospital workers in charge of patients’ care, and healthy controls undergoing health check-up were all present at the same hospital in a limited time period of 12–13 months.

What we found was a considerably higher exposure of hospital personnel to BDV infection compared to unrelated healthy people, in line with a smaller previous report [16]. In our study, the CIC prevalence of medical staff (21.8%) was similar to that of MDD patients (18.2%), and double that of healthy controls without contact with MDD patients (11.1%). Here, one-third of the psychiatry and oncology department personnel (the departments with the highest CIC prevalence) were found to be infected, even though inter-department differences were not significant. Patients in these departments may shed virus more frequently due to elevated virus activation. Immune-competent hospital staff may be able to cope with an elevated exposure to infections as well as high stress levels which in turn promote BDV activation [14]. Then again, sub-clinically infected personnel are a potential source of keeping BDV circulating in hospitals. Where they have an impaired immune system, BDV-infected hospital personnel are exposed to their elevated health risks.

Our study confirms that plasma-based CICs were not only superior to antibody detection but also to PBMC-based BDV p24 RNA for monitoring BDV infection. The majority of subjects were CIC positive with no detectable PBMC-based BDV RNA, indicating antigen release into the plasma (antigenaemia) and CIC formation after primary viremia, consistent with the often discordant presence of serological and RNA markers [6,7]. This disparity is due to over-expressed viral proteins (N/P) vs. a low replication rate of viral RNA [22], which also explains why the highly sensitive RT-qPCR method is less effective in BDV infection monitoring than antigen-driven CIC [2].

Based on more than 1500 subjects in a hospital setting of a Chinese megacity, we found a considerable BDV infection risk among hospital personnel and an association between BDV infection and Chinese MDD patients. Compared to Europe and the Middle-East [2,7,11,21], our study revealed a lower prevalence of BDV infection in healthy people in Chongqing (Southwest China), which increased as a function of age. Unknown transmission of BDV during health care activities which put medical personnel at risk are alarming findings that warrant further serious attention through infection monitoring and preventive measures in China and elsewhere. Our study may alert BDV researchers to conduct further epidemiological surveys, identify transmission routes, and further clarify how BDV infection is linked to psychiatric disorders.
